# Texas hospitals with higher health information technology expenditures have higher revenue: A longitudinal data analysis using a generalized estimating equation model

**DOI:** 10.1186/s12913-016-1367-9

**Published:** 2016-04-05

**Authors:** Jinhyung Lee, Jae-Young Choi

**Affiliations:** Department of Economics, Sungkyunkwan University College of Economics, Seoul, Republic of Korea; Program in Healthcare Management, Hallym University College of Business, Kangwon-do, Chuncheon, 200-702 Republic of Korea

**Keywords:** Health IT expenses, Hospital revenue, Generalized estimation equation, Clustering error

## Abstract

**Background:**

The benefits of health information technology (IT) adoption have been reported in the literature, but whether health IT investment increases revenue generation remains an important research question.

**Methods:**

Texas hospital data obtained from the American Hospital Association (AHA) for 2007–2010 were used to investigate the association of health IT expenses and hospital revenue. The generalized estimation equation (GEE) with an independent error component was used to model the data controlling for cluster error within hospitals.

**Results:**

We found that health IT expenses were significantly and positively associated with hospital revenue. Our model predicted that a 100 % increase in health IT expenditure would result in an 8 % increase in total revenue. The effect of health IT was more associated with gross outpatient revenue than gross inpatient revenue.

**Conclusion:**

Increased health IT expenses were associated with greater hospital revenue. Future research needs to confirm our findings with a national sample of hospitals.

## Background

The Health Information Technology for Economic and Clinical Health (HITECH) Act of 2009, part of the American Reinvestment and Recovery Act, allocated nearly $29 billion over ten years for the implementation and meaningful use of electronic health records (EHRs) throughout the United States (U.S.) health care delivery system. Most of this budget will be spent to incentivize the adoption of health information technology (IT). Hospitals that satisfy meaningful use criteria can receive $2–10 million, and these funds have already been used to support health IT adoption for eligible providers since 2011.

A great deal of prior research has demonstrated the potential benefits of adopting health IT in the hospital setting. These benefits include elimination of duplicate or unnecessary tests and adverse drug events, conserving healthcare provider time and effort by making information more readily available, and cost savings associated with increased efficiency or productivity metrics [1]. A recent economic evaluation meta-analysis of health IT found that nearly 70 % of studies demonstrated value for money of health IT [2]. Other reviews have also shown positive results in specific aspects of medication management [3–7] and chronic disease management such as preventive care and reminders [8–12].

Despite numerous attempts to reveal benefits from health IT, most previous studies on the economic benefits of health IT adoption have focused on cost reduction in the care of individual patients. However, an important managerial question that remains unanswered: does health IT adoption increase hospital revenue? While several studies have demonstrated the positive effects of health IT on revenues through an increase in patient days, outpatient volume [13, 14] and in reimbursement rates through contractual changes [15], they were either single-site or simulation studies.

Health IT can increase hospital revenues in many ways, including reductions in hospital stay, redundant tests, medical errors and administrative expenses [16, 17]. Indeed, previous studies have found a positive association between health IT and hospital performance [18–21]. However, findings from prior studies examining the effects of health IT on costs have been inconsistent, which may result from their use of different data sets and different IT applications. Similarly, several single-site studies have explored the association between health IT and health care costs [22, 23], and they also have reported inconsistent findings. Recently, researchers have examined the relationship between IT adoption and hospital-level costs using the large samples of hospitals [24–26], but, similarly, they reported mixed findings. In one study, Borzekowski [24] used a complete census of 3000 U.S. hospitals with more than 100 beds each from 1987 to 1994 to examine the relationship between health IT use and hospital operating costs. The study used multiple data sources: hospital cost reports (a minimum dataset), the American Hospital Association Annual Survey of Hospitals and the Healthcare Information Management and Systems Society (HIMSS). The study found that both financial/administrative and clinical IT systems at most automated hospitals were associated with lower costs three to five years after adoption.

Furukawa et al. [25] used an unbalanced panel of 326 general acute care hospitals in California to examine the effects of electronic medical records (EMRs) on length of stay, hospital cost, nurse staffing and nurse-sensitive patient outcomes for 1998–2007. They found that EMR use was associated with longer length of stay, higher cost per discharge and lower rates of in-hospital mortality. In terms of nurse staffing, they found that EMRs increased registered nurse hours per patient day and reduced licensed vocational nurse cost per hour.

DesRoches et al. [26] also explored the effect of EMR adoption on quality, efficiency, and cost using cross-sectional data, including the American Hospital Association (AHA) hospital IT survey and annual survey, Hospital Quality Alliance database and The Medicare Provider Analysis and Review. The work included a national sample of 2952 hospitals and cost was measured by the observed-to-expected cost ratio. The study did not find a significant association between EMR adoption and quality of care, efficiency or cost.

Other studies examined the effect of health IT on cost at the discharge level. Amarasingham et al. [27] examined the cross-sectional association between the level of automation measured by physicians’ daily interaction with the IT system and inpatient mortality, complications and cost. They found that hospitals with an automated IT system had fewer complications, lower mortality rates and lower costs, with no difference in length of stay.

To the best of our knowledge, this study is the first to examine the effects of health IT expenses on hospital revenue. To this end, the present study investigates the effects of health IT expenses on revenue by using a large sample of hospitals in Texas from 2007 to 2010. In this study, we selected the state of Texas because Texas has a population of 25.1 million and is the second-largest U.S. state in population just after California. Also, Texas experienced the highest population growth for any U.S. state between 2000 and 2010. However, the basic EMR adoption rate in Texas is 34.8 %, one of the lowest rates among U.S. states [28, 29]. Thus, if there is empirical evidence of the positive relationship between health IT adoption and hospital revenue, it will encourage hospitals to adopt health IT systems and eventually improve quality of care.

To explore the possibility of differential effects on revenue among intra-hospital settings, we modeled the effect of health IT expenses on three different measures of revenue: total revenue, inpatient revenue and outpatient revenue. We made two main contributions to the healthcare IT field. The first contribution of our study is our measure of health IT. Most prior research on health IT has focused on specific applications, such as EMR. Given that there are more than 50 IT applications in hospitals in our sample (HIMSS), focusing on specific IT applications may result in a biased estimate in health IT investment because multiple health ITs have a complex relationship with one another. Thus, we measured IT expenses as the dollar amount given on a hospital’s financial report. The second contribution is the methodology we employed the generalized estimation equation (GEE) model with an independent error component to control for clustered error within hospitals.

## Methods

### Data source

The data used to investigate the effects of investments in health IT on hospital revenue were Texas hospital data from the Center for Health Statistics of the Department of State Health Services for the 2007–2010 time period. Texas hospital data is a subset of the AHA Annual Survey. The AHA Annual Survey profiles more than 6500 hospitals throughout the U.S. AHA data are used by government agencies, the media and the industry for accurate and timely analysis and decision-making [22]. This database has also been widely used in the literature on health IT [30–34]. The database contains hospital-specific data on most Texas hospitals and health care systems (except federal government hospitals), including location, size, structure and personnel. Moreover, these data contain hospital financial information such as revenue, total assets, liability and health IT investment. As we focused our analysis on short-term, acute general hospitals, we excluded psychiatric hospitals, rehabilitation hospitals and government hospitals because they serve unique populations. This study did not require approval from the institutional review board because the subjects in the study were hospitals, not individuals.

### Dependent variables

We used three dependent variables as measures of hospital revenue: total revenue, gross inpatient revenue and gross outpatient revenue. Total revenue was defined as the sum of net patient revenue, tax appropriations, other operating revenue and non-operating revenue. Net patient revenue was estimated from net realizable amounts from patients, Medicaid disproportionate share payments, third-party payers and others for services rendered. That is, net patient revenue stems solely from patient services. Tax appropriation is a predetermined amount from the hospital’s taxing authority to support hospital operation. Other operating revenue is revenue from services other than health care provided to patients, as well as sales and services to non-patients. Lastly, non-operating revenue includes investment income, extraordinary gains and other non-operating gains. Gross inpatient revenue is the hospitals’ full-established rates for all services rendered to inpatients, while gross outpatient revenue is the hospitals’ full-established rates for all services rendered to outpatients.

### Independent variables

As key independent variables, we merged IT capital expenses and IT operating expenses into total health IT expenses. We merged these two variables for several reasons. First, they were complement goods, which means they were goods that were used in conjunction with other goods. Second, as the correlation between them was around 70 %, we would have had a multi-collinearity problem if we simultaneously kept these two variables in our regression. Third, IT capital and IT operating expenses accounted for a very small part of total revenue: only 0.8 and 2 %, respectively. Thus, we could not find any effect of the separate variables on revenue. IT capital expenses include the current year’s IT-related capital and the total value of capital leases to be signed in the current year. IT operating expenses include expenses related to IT operation, but exclude department depreciation and operating dollars paid against capital leases. This measure of IT expenses was a flow variable and different from other IT stock measures employed in other studies [21, 35, 36]. A flow variable is measured over an interval of time (i.e., a year), while a stock variable is measured at one specific time, and represents an accumulated quantity existing at that time.

Based on prior literature, we controlled for an extended set of variables that have been found to be related to revenue including utilization of hospital services (i.e., inpatient days, outpatient visits, emergency visits and government admissions), structural characteristics of hospitals (i.e., bed size, multi-hospital system, ownership and teaching status) and environmental characteristics (i.e., market competition) [30, 37–40].

Inpatient days are the period of service between admission and discharge. This measure includes neonatal and swing days, but not newborns. Outpatient visits are visits by patients who are not lodged in the hospital while receiving medical, dental or other services. Each appearance of an outpatient in each unit constitutes one visit, including all clinic visits, referral visits, observation services, outpatient surgeries, home health service visits and emergency room visits. Emergency room visits reflect the number of visits to the emergency unit. Government admissions include admissions for Medicare, Medicaid and other government admissions such as local admissions, state admissions (such as from Children with Special Health Care Needs) and admissions from the Civilian Health and Medical Program of the Uniformed Services. Government admissions are important because those tend to be more severe and require more intensive treatment than non-government admissions [41].

Bed size was defined as the total number of licensed beds that were authorized by the state licensing agency. ‘Multi-hospital system’ was a dummy variable indicating system affiliation as reported in the Texas hospital data. Ownership was categorized as for-profit (reference), not-for-profit or government. ‘Teaching hospital’ was a dummy variable indicating teaching status.

Market competition was measured using the Herfindahl Index (HHI) based on adjusted discharge. The HHI is an economic concept widely used as to measure competition [42–46]. To compute market competition, first, the adjusted discharge was calculated by summing the inpatient days and outpatient visits for each hospital. Second, the share of adjusted discharge for each hospital for each county was calculated. Lastly, the share of adjusted discharge was squared and summed by county to obtain the market competition or the HHI. Other studies also used the HHI calculated at the county level [43–45].

All cash flow measures were inflated by the Consumer Price Index (CPI) to reflect 2010 U.S. dollars. The study sample included 1493 pooled observations representing 382 unique acute care hospitals in Texas operating between 2007 and 2010. This is an unbalanced panel, which means not all hospitals were observed consecutively over the sample period. Some incomplete cases were included because dropping them may have resulted in biased estimates and longitudinal analysis can control for this unbalanced panel data.

### Analysis

To understand the effect of health IT expenses on hospital revenue, we modeled the effect of hospital health IT expenses on total revenue controlling for observable hospital utilization, hospital and market characteristics such as inpatient days, outpatient and emergency visits, government admission, bed size, system membership, ownership, teaching status and competition. Formally, we estimated regressions based on the following specification:

1 $$ \begin{array}{l}\ {\mathrm{Revenue}}_{\mathrm{i}\mathrm{jt}}={\upalpha}_{\mathrm{i}}+{\upbeta}_1\mathrm{Inpatient}\ {\mathrm{days}}_{\mathrm{i}\mathrm{jt}}+{\upbeta}_2\mathrm{Outpatient}\ {\mathrm{visits}}_{\mathrm{i}\mathrm{jt}}+{\upbeta}_3\mathrm{Emergency}\ {\mathrm{Visits}}_{\mathrm{i}\mathrm{jt}}\\ {} + {\upbeta}_4\mathrm{Government}\ {\mathrm{Admissions}}_{\mathrm{i}\mathrm{jt}}+\updelta\ \mathrm{IT}\ {\mathrm{expenses}}_{\mathrm{i}\mathrm{jt}}+{\upgamma}_1\mathrm{Licensed}\ {\mathrm{Beds}}_{\mathrm{i}\mathrm{jt}}+{\upgamma}_2\mathrm{System}\ {\mathrm{Member}}_{\mathrm{i}\mathrm{jt}}\\ {} + {\upgamma}_3{\mathrm{Government}}_{\mathrm{i}\mathrm{jt}}+{\upgamma}_4{\mathrm{NFP}}_{\mathrm{i}\mathrm{jt}}+{\upgamma}_5{\mathrm{Teaching}}_{\mathrm{i}\mathrm{jt}}+{\upgamma}_6{\mathrm{HHI}}_{\mathrm{i}\mathrm{jt}}+{\uptheta}_1\mathrm{Time}+{\uptheta}_2{\mathrm{Time}}^2+{\in}_{\mathrm{i}\mathrm{jt}}\end{array} $$

where *i* represents hospital, *j* represents county and ∈ _*ijt*_ represents random error. Parameters *β*_1_ through *β*_4_ capture utilization. The parameter *δ* captures the change in revenue with health IT expenses. The *γ*_1_ through *γ*_6_ parameters measure the effects of the hospital and market characteristics. Lastly, the time function is approximated by a second-order polynomial to measure the non-linear effect of time, *θ*_1_ and *θ*_2_.

The measures of revenue and health IT expenses were all financial variables, which means that there may be a correlation problem that could lead to biased estimates. To control for this possible problem, we employed a generalized estimation equation (GEE), which is most suitable with longitudinal data. One of the strengths of GEE is that it does not require correct specification of the distribution, but only of the mean structure [47]. Also, another strength of GEE is the selection of variance structure, which plays an important role in controlling the correlation of longitudinal data analysis. A number of different variance covariance structures are available that cover assumptions of the associations between responses. For example, an independent covariance structure would be best fit if none of the responses are correlated. An exchangeable covariance structures would be appropriate when responses from the same cluster are equally correlated. An autoregressive covariance structure would be appropriate when the correlation between responses decrease with time. Lastly, an unstructured covariance structure would be appropriate when the correlation between responses is comparatively complex [48]. The criteria used to identify best appropriate covariance structure gives the best trade-off between model fit.

Thus, we applied GEE to control variance structure and control clustering error within hospitals. For model selection, we tested the quasi-likelihood under the quasi-information criterion (QIC) and chose the independent variance-covariance matrix with the smallest QIC among many possible variance structures such as unstructured, exchangeable, stationary, AR(1) and AR(2) [49]. All statistical analyses were conducted using STATA software (version 11.0; STATA Corporation, College Station, TX).

## Results

**Fig. 1:**
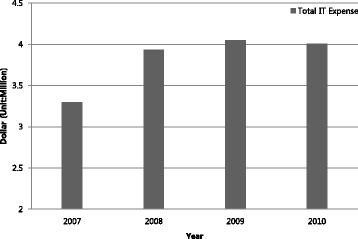
Health IT expenditure per year Trend from 2007 to 2010 (Unit: Hospital)

Table 1 describes hospital utilization, hospital characteristics and market characteristics. The average number of inpatient days was 32,751, while the number of outpatient visits was almost three times larger. Emergency visits and government admissions were 24,865 and 4089, respectively. The average number of licensed beds was 179. The total health IT expenses were around $3.7 million per hospital over a four-year period. For more detailed information, we tracked health IT expenditure from 2007 to 2010 in Fig. 1. This indicates that health IT expenditure significantly increased from 2007 to 2008, but decreased slightly from 2009 to 2010. For-profit hospitals accounted for 34.3 % and not-for-profit hospitals accounted for 30.9 %. More than one fifth of the hospitals in our sample were non-teaching hospitals. Competition measured by the HHI was 54.9 %. The average total revenue was $127 million, gross inpatient revenue was $230 million and gross outpatient revenue was $143 million.

**Table 1:** Descriptive statistics of a sample of 1493 pooled observations representing 382 unique acute care hospitals

Variable	Mean	Std. Dev.
Inpatient days	32,751	50,415
Outpatient visits	90,674	169,451
Emergency visits	24,865	28,913
Government admissions	4089	5813
Total IT investment ($)	3,674,762	9,561,710
Number of Licensed Beds	179	242
System member	26.9 %	
Ownership		
For profit	34.3 %	
Government	30.9 %	
Not for profit	34.8 %	
Teaching Status	22.0 %	
Competition (HHI)	54.9 %	37.6 %
Total Revenue ($)	127 mil	206 mil
Gross Inpatient Revenue ($)	230 mil	388 mil
Gross Outpatient Revenue ($)	143 mil	197 mil

Table 2 presents GEE regression results with an independent variance-covariance matrix. We employed three measures of revenue as dependent variables (total revenue, gross inpatient revenue and gross outpatient revenue) because health IT expenses may affect revenue differently across inpatient and outpatient settings. The left-most column shows the effect of health IT expenses on total revenue. We found that health IT expenses were significantly and positively associated with total revenue. Specifically, total revenue was expected to increase by 8 % when health IT expenses increased by 100 %. We also found that hospital utilization including inpatient days and outpatient visits was positively associated with total revenue. However, other utilization such as emergency visits and government admissions was negatively associated with total revenue. Other hospital characteristics that were significantly and positively related to total revenue included teaching status and a more competitive environment.

**Table 2:** GEE regression results with independent variance-covariance matrix: a sample of 1493 pooled observations representing 382 unique acute care hospitals in Texas operating between 2007 and 2010

Variables		Total revenue	Gross inpatient revenue	Gross outpatient revenue
		Coefficient (Std. Err)	Coefficient (Std. Err)	Coefficient (Std. Err)
Inpatient days (in log)		0.874** (0.037)	1.218** (0.037)	0.359** (0.043)
Outpatient visits (in log)		0.190** (0.012)	−0.008 (0.015)	0.273** (0.014)
Emergency visits (in log)		−0.051** (0.014)	−0.136** (0.014)	−0.073** (0.016)
Government admissions (in log)		−0.122** (0.028)	−0.208** (0.029)	0.323** (0.034)
Total IT investment ($) (in log)		0.080** (0.008)	0.047** (0.008)	0.058** (0.009)
Number of Licensed Beds (in log)		−0.024 (0.033)	−0.028 (0.032)	−0.089* (0.034)
System member		−0.005 (0.012)	−0.034** (0.013)	−0.084** (0.015)
Ownership	Government	0.008 (0.022)	−0.619** (0.028)	−0.232** (0.026)
	Not for profit	0.044 (0.015)	−0.202** (0.014)	−0.041* (0.017)
Teaching status		0.047 ** (0.011)	0.046** (0.012)	−0.021 (0.014)
Competition	HHI	−0.492** (0.037)	−0.345** (0.041)	−0.199** (0.038)
Time effects	Time	0.072** (0.026)	0.078** (0.028)	0.119** (0.034)
	Time^2^	−0.004 (0.005)	0.000 (0.005)	0.002 (0.006)
Constant		7.908** (0.183)	9.408** (0.176)	9.563** (0.194)

** p < 0.01, * p < 0.05

In the second and third columns of Table 2, we compared the effect of IT on gross inpatient and outpatient revenue. The magnitude of the effect was greater for gross outpatient revenue than for gross inpatient revenue. Specifically, when health IT expenses increased 100 %, gross inpatient and outpatient revenue were estimated to increase by 4.7 and 5.8 %, respectively.

Hospital utilization showed different patterns of gross inpatient and outpatient revenue. For example, the inpatient days were more associated with gross inpatient revenue than with gross outpatient revenue. However, the outpatient visits were only associated with gross outpatient revenue. Other utilizations including emergency visits and government admissions were differently associated with gross inpatient and outpatient revenue. Also, hospital characteristics play roles in deciding gross inpatient and outpatient revenue; system members, ownership such as government and not-for-profit and more competition were positively associated with gross inpatient and outpatient revenue.

## Discussion

We examined the effects of health IT expenses on revenue at the hospital level using Texas AHA data from 2007 to 2010. The GEE model was employed to control for variance-covariance error in financial information and clustering error within hospitals. We found a significant and positive effect of health IT expenses on hospital revenue. Our findings are consistent with those of previous studies [13, 21, 50]. Health IT makes patient information easily accessible where it is needed, supports better health care decision making, promotes better coordination of care, increases processing speed and eliminates duplicate or unnecessary tests [51]. Accordingly, health IT may result in increased revenue in a hospital setting. The most evident explanation for this includes improved capabilities facilitated by health IT such as an EMR system that allows capture of previously lost revenue by eliminating inefficiency [13].

As expected, we found that hospital utilization showed difference pattern on hospital revenue. Specifically, while inpatient days and outpatient visits were positively associated with revenue, emergency room visits and government admissions were negatively associated with revenue. This suggests that hospitals lose money on emergency room visits and government admissions [52]. Patients admitted to the emergency room are cared for regardless of their ability to pay because emergency care is the last safety net. Emergency physicians thus provide uncompensated care to uninsured patients, which may reduce hospital revenue [53]. Also, a recent study found that the bad debt provision, as the one component of uncompensated care was negatively associated with operating margin among private non-profit hospitals [37]. Moreover, the reimbursement rate from government admissions has been reported to be less than that of private insurers; private insurers reimburse an average of $1226 for low-back disc surgery, while Medicare will only reimburse $654 [53].

Hospital characteristics also played a significant role in hospital revenue. Teaching status and a competitive environment were important factors in modeling total revenue. We found that teaching status was positively associated with hospital revenue. Moreover, a more competitive environment was positively associated with hospital revenue, suggesting that competition may improve efficiency, which can lead to improved revenue generation.

Moreover, we investigated whether the effect of health IT expenses differs between inpatient and outpatient settings. We found that health IT had a stronger association with gross outpatient revenue than inpatient revenue. Prior studies using HIMSS data focused on inpatient stays because EMR adoption information was available only for inpatients, not for outpatients. However, by utilizing all IT expenses in the hospitals in our model, we revealed that health IT expenses were more associated with the outpatient setting revenue. Compared to the inpatient setting, the outpatient setting is more complex with numerous procedures, surgeries and tests [54]. It is important that information is acquired quickly, there is efficient feedback about appropriateness of the procedure and costs of medication is readily accessible. Another explanation is related to the current trend of shifting inpatient procedures to outpatient procedures [54]. With this prevailing trend, hospital health IT behavior might be optimized in the outpatient setting where the effect of health IT on revenues is greater.

There are several limitations to consider when interpreting our findings. The first limitation pertains to the limited external validity as we used data from hospitals in Texas. We are, therefore, unable to generalize our findings to other states with different patterns of IT expenses or other countries with different hospital financing systems. Second, for the purposes of this study, ‘health IT expenses’ were broadly defined as dollars invested in both capital and operations related to IT. Also, our measure of IT expenses was a flow variable, not a stock variable, that was measured as the accumulated quantity existing at that time. Thus, if we used stock variables as were used in a previous study [21], the effect of IT capital on revenue would be larger. Moreover, while we included IT operating expenses in the IT expense measure, certain costs of health IT, like backfill time for IT personnel, management or workflow redesign, could not be measured. However, the time period we selected occurred before the HITECH act, which means that EHR adoption rate is low (around 1–2 % of revenue) [21, 30]. Thus, unmeasured costs of health IT spending may be trivial. Third, IT expenses may differ in a context that we could not observe in the data, such as quality or efficiency. In this case, the estimators may remain biased. Fourth, there may be reverse causality between revenue and health IT investment. For example, hospitals that expect more revenue may have been more likely to invest in health IT. Thus, in this case, our estimates may have been biased. Fifth, the associations we are investigating are likely affected by the baseline status of health IT; however, we could not access this data. Thus, this potential association between health IT expenses and revenue may change over time depending on EHR implementation within those systems. Sixth, the time period of data collection was just before the implementation of the HITECH act. Thus, the results of our study may be different from the current period or the period just after implementation of the HITECH act. Hospitals did not have much incentive to adopt health IT systems before the HITECH act, while they were recommended to adopt health IT systems by the HITECH act. Last, but not least, there may be unobserved confounding factors that might impact our estimates; for example, organizational and management behavior may be correlated with IT investment [24]. Many barriers for adopting IT system were reported; workflow disruption, communication among users, complexity, physical space and resistance from physicians [30, 55]. Thus, these barriers could reduce the effect of IT system on revenue even if IT system was adopted in the hospitals.

## Conclusion

The current work investigates the association between health IT adoption and hospital revenue. We filled a gap in the literature concerning the study of health IT adoption by revealing the beneficial effects of health IT expenses on hospital revenue. An important finding is that hospitals that incurred greater health IT expenses tended to generate more revenue, and the magnitude of the observed association was particularly large in the outpatient setting of hospitals. Future research is needed to confirm our findings using a national sample of hospitals.
